# Biomechanical Evaluation of Exercises for Performing a Forward Handspring - Case Study

**DOI:** 10.2478/v10078-012-0060-2

**Published:** 2012-10-23

**Authors:** Kamenka Živčić-Marković, Goran Sporiš, Ines Čavar, Aleksandra Aleksić-Veljković, Zoran Milanović

**Affiliations:** 1 Faculty of Kinesiology, University of Zagreb, Zagreb, Croatia.; 2 Faculty of Sport and Physical Education, University of Nis, Nis, Serbia.

**Keywords:** gymnastics, methodology, basic exercises, biomechanical analysis

## Abstract

The aim of this study was based on the kinematic parameters, extracted at different stages of performing a forward handspring to determine the interconnection of methodological procedures of learning with the final structure of the movement. The respondent is an active competitor with years of experience, elite athlete, many times Croatian champion, and competitor at European, World Championships and the Olympics. The team composed of six gymnastic experts, chose one of the best performances by twelve methodological procedures and the best performance (of six) two-leg forward handsprings basing their choice on a detailed review of recorded material. Assessment of quality of performance was done according to the defined rules prescribed by the regulations (Code of Points). The forward handspring technique consists of four phases based on which 45 space and time kinematic parameters were selected (30 parameters in the phase of hand contact and push-off, 7 in the flight phase, and 8 parameters in the landing phase). By extraction of space and time parameters, there was a differentiation of certain methodological procedures that are the best for learning forward handspring in each phase of its performance. This research indicates that these methodological procedures mostly coincide in space kinematic parameters by which the technique of a forward handspring is described.

## Introduction

In gymnastics, the forward handspring is one of the key elements of acrobatics from which further connections with other acrobatic elements, with rotation about the frontal axis of the body, are carried out (Karascony and Čuk, 2005; Živčić, 2000; Živčić et al., 2007). This element is an integral part of the run-up in acrobatic series. It can be performed from different initial positions where the main goal is to transform the linear movement of the body to rotational, with minimal loss of horizontal velocity. Also, it is necessary to create the basic preconditions for push off and the successful implementation phases of flight (Živčić, 2000; Živčić, 2007). According to previous theoretical and scientific knowledge, one of the dominant phases in the forward handspring is the hand-surface contact and push-off (George 1980; Karascony and Čuk, 2005; Živčić et al., 2007). It is defined by the angle of body CG (centre of gravity) in relation to the surface, the angle of the shoulder joint and the horizontal and vertical velocity of the body’s CG at the time of last contact with the hand. Since the parabola of flight of the forward handspring is primarily defined by horizontal and vertical velocity, the duration of this phase should be as short as possible, and evident throughout the duration of push-off (Hay, 1985; Knoll, 1996; Prassas et al., 2006).

Based on biomechanical analysis, it is possible to identify the technique (Arampatzis and Brüggemann, 1998), make a comparison of different techniques (Franks, 1993; Knoll, 1996; Yoshiaki et al., 2003; Prassas et al., 2006b), specify errors in performance (Živčić et al., 1996; Nakamura et al., 1999), determine the biomechanical characteristics of the gymnastic apparatus (Daly et al., 2001), evaluate the influence on athlete’s injury prevention (Taunton et al., 1988; Sands, 2000; Self and Pain, 2001; Beatty et al., 2006), and quickly get feedback on key parameters (McGuane, 2002; Beatty et al., 2006; King, 2011). Biomechanical studies generally should be related to the training process and thus enable the creation of rapid and successful interaction between coaches and athletes.

According to our current knowledge, there is insufficient number of biomechanical studies on the methods of training individual gymnastic elements and application of some methodological procedures. Methodological procedures have an important role in coaching a gymnast at any stage of his development. Generally, methodology of training in gymnastics is based on practically proven methods of learning, developed by joint work of coaches and athletes where the terminology, number and order of the methodological procedures was established through years of gymnastic experience (Gwizdek, 1992). From the scientific point of view, methodical basics of learning should be focused on scientific verification which could certainly help in providing precise information on the number of parameters relevant to the performance of each gymnastic element.

The aim of this study, was to determine the interconnection of methodological processes of learning with the final structure of movement.

## Methods

The respondent in this research was an active competitor with years of experience, elite athlete, many times Croatian champion, and competitor at European, World Championships and the Olympics. The respondent, an elite gymnast, with his anthropometric characteristics, fits the championship model (body height: 161 cm, body mass: 59 kg). The Ethics Committee of the Faculty of Kinesiology, University of Zagreb, approved all experimental procedures according to the revised Declaration of Helsinki.

### Measures

A team composed of six gymnastic experts, chose one of the best performances by twelve methodological procedures and finally decided on the best performance (of six) two-leg forward handspring basing their choice on a detailed review of recorded material. Assessment of quality of performance was done according to the defined rules prescribed by the regulations (Code of Points) (FIG, 2006) to determine the quality of performance: the amplitude of body movement and individual body segments, matching the movement of paired segments of the body and the precision of implementation of each phase.

### Sample of variables

**Figure 1:**
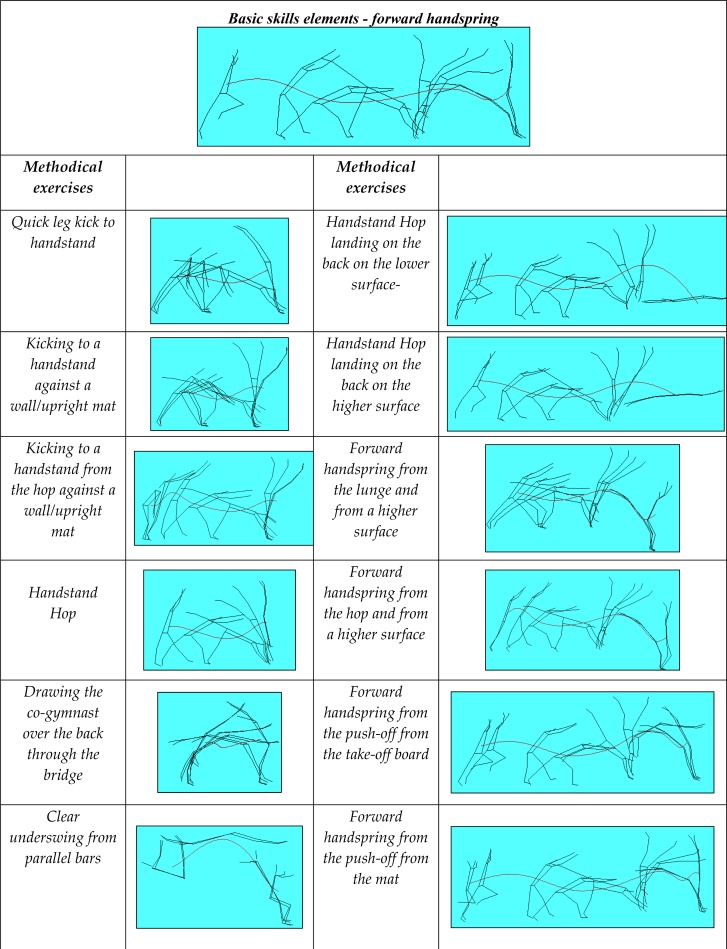
Forward handspring and methodical exercises

*Selection of methodological procedures*. The forward handspring is trained through characteristic stages (Figure 1), and as a whole, but in facilitated conditions, by analytical and synthetic methods of learning. Accordingly, this research selected twelve methodological procedures that could be considered the most appropriate for training the forward handspring.

*Selection of kinematic variables*. The forward handspring technique consists of four phases based on which 45 space and time kinematic parameters were selected (30 parameters in the phase of hand contact and push-off, 7 in the phase of flight, and 8 parameters in the landing phase). For the purpose of precise comparison of results, kinematic parameters that characterize each phase of performance for which the training process is designed were extracted. Based on the general characteristics of the biomechanical performance, 45 parameters were analyzed (Table 1).

### Procedure

A film was made with two VHS (Panasonic NV-MS1 HQ S-VHS) video cameras, at a speed of 60 frames per second. Each of them analyzed the motion at the time of the hands-surface contact, where the cameras were positioned at an angle of 45° to the axis perpendicular to the direction of movement of subjects and passing through the vertical center of the push-off. In two methodological procedures, which aimed at the learning phase of landing, the place was the location of the vertical landing.

Camera lenses were at the hip level of the subject, 2m from the line of performance. All movements were performed in the same direction. Data processing was carried out according to the standards of APAS (Ariel Performance Analysis System, 1995) procedures for the kinematic analysis which included 17 reference points and 15 body segments and conducted through several phases: digitalization of the recorded videos and the reference points of the body, transforming the three-dimensional space, data filtering and calculation of kinematic quantities. The seven segment anthropometric model was also used (foot, shank, thigh, trunk, upper arm, forearm and head) (Miller and Nelson, 1973).

### Statistical analysis

The results, as well as a graphical presentation of the results obtained were analyzed by software package Statistica 7.0 for Windows (Statsoft, Inc.2004, Tulsa, Oklahoma, USA). In order to verify the biomechanical justification of the analyzed methodological procedures for training the forward handspring hierarchical cluster analysis was used (Ward’s method based on the Euklid’s distance). The results are presented in dendrograms which show the entire course of the creation of a hierarchical group of methodological procedures and the level at which an object joins the group on the basis of their analogy.

## Results

At the phase of setting hand-surface contact based on the space parameters, a hierarchical cluster analysis resulted in two homogeneous groups. The first group of methods has the greatest resemblance to the final structure of the motion (handspring) as follows: handstand hop landing on the back on the lower surface, forward handspring from the push-off from the take-off board, forward handspring from the push-off from the mat, handstand hop landing on the back on the higher surface. They are characterized by performing a run-up and hop, and have strong similarities in the values of the parameters specific for this type of performance as opposed to the techniques without the runup. Specific parameters are related to: the duration of the bounding step (0.150 and 0.167 s), CM height after bounding step (90.2 – 91.2 cm), the hip angle in the take-off phase (136–142º) and CM height in the first hand-surface contact (79.6 –82.7 cm, 71.9 cm somersault).

The second group consists of analog methodological procedures that are performed without run-up or hop. In this group the most obvious similarity was observed between the values of the thigh and trunk angles, and upper and lower leg, since it is derived from similar starting position where the angles of the knee and hip joint were maximally open (180°). Also, there are minimal oscillations of the values of two kinematic parameters that determine the quality of execution of this phase, and those are the angles (35°– 38°), similarly as in a handspring (38°), and the height of CG in the first hand-surface contact (61.9 – 65.8 cm). Unlike other groups of elements, the first large differences in values were related to the length and height of body CG in characteristic positions of performance in this phase.

Hierarchical cluster analysis of the methodological procedures and the forward handspring, based on time kinematic parameters of two homogeneous groups were also obtained where it was visible that the formation of analogous groups participated in the same methodological elements as in the previous analysis. Each group was characterized by similar parameters related to horizontal and vertical velocity of body’s CG in the bounding step, the first leg-surface contact and the last contact of the swinging leg, which had very low values compared to the first analog group.

On the basis of space kinematic parameters of the take-off phase two homogeneous groups were obtained. To study this phase of a forward handspring a smaller number of cases was used (8) than in the previous phase. The first group, which was closest to the forward handspring, was most similar to the value of CG in the angle of the body to the ground and shoulder joints, and the height of the CG in the last hand-surface contact, while the second group of elements and values was significantly lower. In the remaining physical parameters, there was no significant difference to discriminate the other two groups.

Unlike the previous analysis of time parameters, there is an apparent difference in the analogous grouping of elements in relation to the forward handspring. In this case, three homogeneous groups were obtained. The first group consists of the methodical procedures that have similar values of horizontal velocity of the body in the take-off phase and duration of take-off phase, unlike other groups that have very similar values of vertical velocity in the take-off phase. An element drawing the co-gymnast over the back through the bridge formed the third group, where it was noticed that in any of the extracted parameters (Table 2), there was no similarity with the remaining methodological procedures, or the final element. Its main purpose is primarily focused on achieving and maintaining proper body position which defines the angles between body segments (Table 2).

In the flight phase, by separations of methodological procedures on the basis of space and time parameters, a visible difference in analog grouping was noticed. In an analysis of the space parameters, three homogeneous groups of elements were obtained. The first group, which consisted of only two elements, had a great similarity in the values of the height and duration of the flight as well as the angles of the knee joints at maximum height. In the second group, the similarity between the individual elements was reflected in the duration of the flight and the angles between body segments at maximum flight, which was consistent with the forward handspring. As in the previous analysis (take-off phase), the third group re-formed drawing the co-gymnast over the back through the bridge. This methodological procedure had no significant similarity in the parameters that determined the flight parabola (length and height of flight) with the remaining variables and forward handspring. During the flight phase similarity is evident in the values of the angles between the upper arm and trunk, as well as between thigh and lower leg

Unlike the hierarchical grouping of elements according to the space parameters, based on time parameters, we created two homogenous groups. The first group, along with a forward handspring, made methodical procedures that were closest to the value of horizontal velocity at maximum flight time. In these parameters there were large differences in relation to the exercise drawing the co-gymnast over the back through the bridge which was further away from the other independently formed group.

In the landing phase, by taxonomy of methodological procedures based on the values of physical parameters, two analogue groups were obtained. The first group was characterized by close values of the physical parameters related to the angle of CT of the body to the surface, the angle of the knee joints and hips in the first foot contact with the surface (Table 2). In the second group of elements there were significantly greater distances than in the first group, while the greatest similarity was observed in the values of the angles of joints of hips and knees throughout the landing phase. By implementation of the hierarchical cluster analysis concerning the time parameters and the landing phase, two homogeneous groups were also obtained. At the closest distance from the forward handspring, this also had the nearest value of the horizontal and vertical speed CT of the body at the time of the first contact with the feet. Drawing the co-gymnast over the back through the bridge also belonged to this group. All remaining elements formed a separate group, characterized by matching the values related to the horizontal and vertical velocity of the body’s CG at the time of landing, which was different compared to the first group (Table 2).

## Discussion

Respecting the planning and programming processes of training in artistic gymnastics which is primarily focused on fast and efficient learning some gymnastic elements with the basic theoretical principles of learning: from easier to more difficult, from simple to complex, from the known to the unknown (Bloom, 1985), we can assume that the learning process itself will be based on the quality and range of applicability of each element of gymnastics in terms of its basic goals and purposes. For this reason, the training process is recommended to be used as a combined method of learning that suggests the use of synthetic methods and, if necessary, analytical (if needed for certain parts of the motion), if there are complex movements from which we can extract many biomechanical parameters such as the handspring.

The results show that the methodical procedure, drawing the co-gymnast over the back through the bridge, has no similarity with the final structure, or with the remaining methodological procedures, due to space and time parameters extracted at the last hand-surface contact. This is because the techniques of performance of this exercise do not imply biomechanical principles that characterize the take-off, but there is a gradual separation of hands from the surface over the fingers. At this point, the body is twisted in a maximum position with a large extension in the shoulder joint (Živčić, 2000; Živčić, 2007). Drawing the co-gymnast over the back through the bridge is an exercise that is performed by a practitioner in constant contact with a co-gymnast, which allows him to perform. Moving back over a co-gymnast, ensures the correct position of the body that should have the final movement phase of the flight. It is achieved according to the extracted parameters related to the angles between the various segments of the body, but there are no similarities according to any other characteristics that define the trajectory of the flight. The methodical process of drawing a co-gymnast over the back through the bridge when the body passes over the back of the co practitioner cannot be characterized only as a flight phase. Therefore, this procedure has the greatest similarities with a forward handspring in the landing phase, especially in the angle and the height of body’s CG during landing, and the angle between upper arm and trunk. The main purpose of this method is to achieve a regular position of the body, which is manifested through the values of physical parameters in the landing phase.

Elements performed with the help of training aids, rather than of the surface form a separate homogeneous group. Their performance is facilitated by the use of springboards. In these processes the most significant methodological differences are between the analyzed variables in the lower CT values of the angle of the body to the ground at the time of taking off. Accordingly, we can observe higher values of vertical velocity of the body’s CG at the time of taking-off caused by the elasticity of the surface. It also illustrates the difference in the values of angles in the shoulder joint at the time of taking off for less than 10° at the forward handspring. With these groups of exercises a significant increase in vertical velocity of the body has been noticed, which at this stage should be of a lower value, and the main reason for this is because the phase of the flight is primarily oriented towards achieving the highest possible level. In these exercises, there have been no significant changes in the position of the body.

After examining the grouping of some exercises that include the phase of the flight, it is possible to notice that the formation of the first homogeneous group has primarily been based on the value of the length and height of the flight parameters that describe the trajectory of the flight. Another homogeneous group, still at a small distance from the first group, has been made according to the individual body segments attained at a maximal flight, which indicates that the performance has been correct.

Based on the characteristics of the flight phase (George, 1980; 2010) which is defined by the kinematic parameters such as height, length and duration of the flight, it is evident that there has been a matching of values in most methodical procedures with the final structure of movement. The greatest similarity is between the values of physical parameters related to the angles between body segments. Because the main purpose of the analyzed exercises is to reach correct positions of the body at the flight phase, it may be considered that, in spite of the method and terms of exercise performance, there has been matching in execution of the flight phase during forward handspring. Great similarities are also visible in the parameters related to the horizontal velocity of the body CG at all methodical exercises except exercise drawing the co-gymnast over the back through the bridge.

In the phase of the flight, which is primarily oriented to the length and height of the flight, it is very difficult to accomplish the requirements of the space and time parameters in terms of their mutual compatibility by the presentation of certain methodological procedures. With the respect to the position of the body defined by relationships between the individual body segments and the angles of the joint system, it is evident that the exercises that involve a flight phase have the greatest similarity in these parameters. Similarities in the time parameter at this stage, due to hierarchical clustering, are obviously not caused by the initial position, but the preconditions for a successful flight phase are formed during the hand-surface contact and push-off.

The closest grouping has been noticed at methodological procedures that have slight differences in the values of horizontal velocity of the body’s CG at the maximal flight. Somewhat larger differences appear in the horizontal velocity of CG at the maximal flight, in relation to this set of procedures, observed in a forward handspring from the hop and from a higher surface, but similar to a forward handspring.

Considering the duration of the flight, methodical procedures which are grouped into a homogenous group with a forward handspring have similar values, but are more similar to the whole structure, except for a forward handspring from the push-off from the take-off board which has a similar time to the value of the forward handspring. The exercise drawing the co-gymnast over the back through the bridge, which makes a separate group, very distant from the first hierarchical group of processes and the final element, differs significantly in the time parameters that define the phase of the flight. The duration of the flight with this procedure is three times longer than at the other procedures, and has a very low value of the horizontal velocity of the body’s CG during the maximal flight.

Based on the biomechanical characteristics of the key phases of landing (Self and Panels, 2001; McNitt-Gray et al., 2005; Lilly et al., 2007; George 2010), and the previous analysis, it may be noted that at the time of the first foot contact with the surface there are similarities in physical parameters of the forward handspring, and they refer to the angles between body segments (upper arm and trunk, upper legs and trunk, and upper and lower leg) in the majority of exercises that involve landing. The correctness of the position of the body is characterized by the level of performance in landing (Živčić and Omrčen, 2009). It may be concluded that most exercises which include the landing phase, meet the basic requirements prescribed.

It is also noticeable through the analysis that the procedures which are not done from the raised surface have very close values of the angle and height of body’s CG at the time of the first contact with the surface. The procedures which are performed from the raised surface have a greater height obtained in the flight phase, and thus higher values of these parameters in the landing phase.

The value of the velocity of the body’s CG at the time of landing is much closer to horizontal velocity of body’s CG in methodological procedures that are performed from raised surfaces, as opposed to vertical velocity extracted at the same time, which are two to three times higher in methodological procedures, than at the forward handspring (Živčić and Omrčen, 2009).

## Conclusion

By taxonomic analysis of biomechanical parameters of methodological procedures for learning a forward handspring, it can be argued that an expert analyzed action is justified, and thus relevant for use in teaching of the mentioned element with selected young gymnasts. By extraction of space and time parameters, there was a differentiation of certain methodological procedures that are best for learning the forward handspring in each phase of its performance. This research has determined the fact that these methodological procedures mostly coincide in space kinematic parameters by which the technique of performing the forward handspring is described.

## Figures and Tables

**Figure 2:**
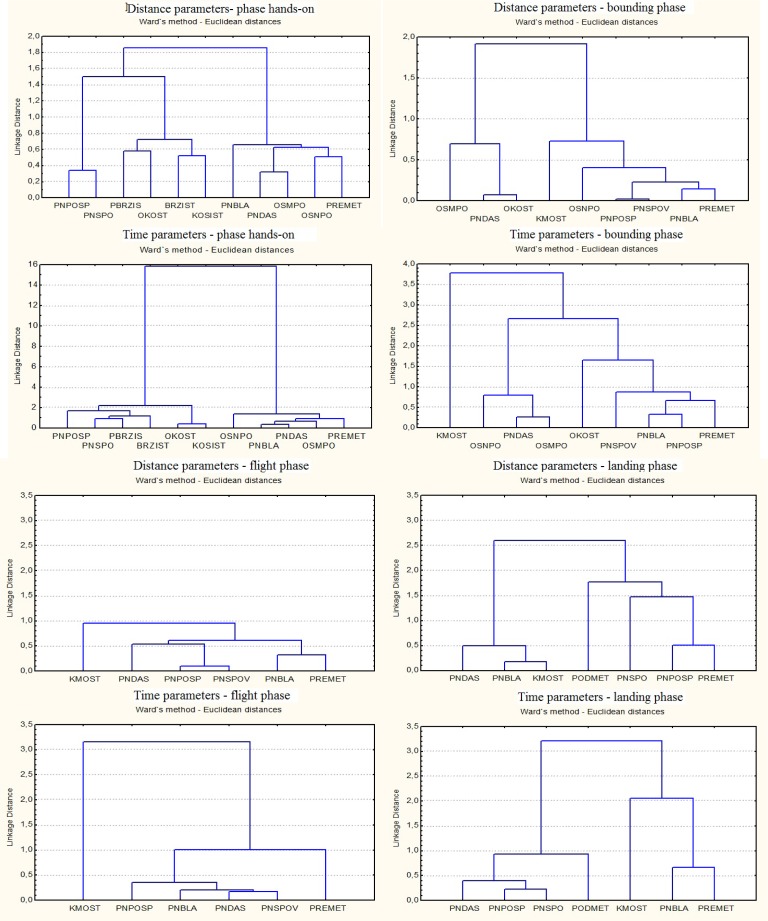
Dendograms for all parameters

**Table 1:** Longitude, altitude, time and angles

	**Forward handspring**	**QLKH**	**KHAWM**	**KHHWM**	**HH**	**HHLS**	**HHHS**	**CUPB**	**BRIDGE**	**FHLHS**	**FHHHS**	**FHPBS**	**FHPOM**

Lunge length (cm)	96.1	82.4	69.1	92	90.7	85.8	107.1			97.4	94.5	87.8	97.3
Lunge time (sec)	0.18	0.11	0.13	0.26	0.51	0.15	0.16			0.13	0.20	0.15	0.16
CG height after bounding step (cm)	90.7	76.4	79.1	99.4	91.7	90.9	91.2			81.4	98.3	90.2	91
CG height in first contact take-off leg (cm)	68.4	73.1	73.1	75.2	76.2	73.2	70.1			73.7	69.3	71.2	70
Hand-feet distance (cm)	91.8	113.5	85.9	101	105.8	117.3	112.3			87.3	89	103	101
CG height in first hand-surface contact (cm)	71.9	61.9	63.3	65.8	65.3	79.6	71.1		79	65.4	65.2	82.7	76.4
CG height in push-off phase (cm)	90.2				90.4	98.3	98.9		79	88.5	89.5	83.5	88
Push-off phase time (sec)	0.23				0.21	0.25	0.22		1.17	0.25	0.32	0.13	0.28
Max CG height in flight phase (cm)	93.2					132	109.8		75	89.0	91.6	108.6	91
Flight phase length (cm)	74.7								27	64.3	84.1	91.5	51.3
Flight phase time (sec)	0.30								0.98	0.42	0.43	0.47	0.28
Height of the body’s CG at the moment of landing (cm)	75							75	74	92	91.7	82.8	77

	**Forward handspring**	**QLKH**	**KHAWM**	**KHHWM**	**HH**	**HHLS**	**HHHS**	**CUPB**	**BRIDGE**	**FHLHS**	**FHHHS**	**FHPBS**	**FHPOM**

Knee angle in swing leg after bounding step (rad)	174	190	184	186	192	175	169			187	184	170	174
Knee angle swing leg in last contact with surface (rad)	182	187	185	185	195	186	184			190	188	184	179
Knee angle swing leg during swing phase (rad)	152	174	166	165	150	150	154			134	140	142	143
Knee angle take-off leg in take-off phase (rad)	184	177	171	195	172	184	194			183	189	186	164
Min knee angle take-off leg (rad)	144	125	129	138	124	136	142			120	123	140	138
Hip angle swing leg after bounding step (rad)	151	171	166	171	181	149	144			186	173	147	154
Hip angle swing leg during swing phase (rad)	159	147	153	161	165	163	164			174	166	162	162
Hip angle take-off leg in first contact after bounding step (rad)	72	113	99	97	90	89	80			94	79	82	77
Hip angle take-off leg at take-off moment (rad)	82	86	75	94	80	104	100			94	95	96	81
Shoulder angle in first hand-surface contact (rad)	137	148	135	140	145	134	148		185	117	121	136	142
CG angle in first hand-surface contact (rad)	38	37	37	35	37	30	43			38	35	41	27
CG angle in push-off phase (rad)	102			69		85	93		119	105	106	70	95
Shoulder angle in push-off phase (rad)	165				144	139	156		188	141	120	151	169
The shoulder angle in maximal flight (rad)	213					165	177		207	191	189	208	189
The knee angle in maximal flight (rad)	173					190	193		188	186	185	184	176
The hip angle in maximal flight (rad)	213					191	195		231	204	212	216	223
The knee angle at the moment of landing (rad)	45							60	44	65	68	47	43
The shoulder angle at the moment of landing (rad)	201							20 3	211	197	189	187	209
The angle of the body’s CG in at the moment of landing (rad)	190							17 3	227	180	188	220	222

CG- center of gravity; QLKH- quick leg kick to handstand; KHAWM- Kicking to a handstand against a wall/upright mat; KHHWM- Kicking to a handstand from the hop against a wall/upright mat; HH- Handstand Hop; HHLS- Handstand Hop landing on the back on the lower surface; HHHS- Handstand Hop landing on the back on the higher surface; CUPB - clear underswing from parallel bars; BRIDGE- Drawing the co-gymnast over the back through the bridge; FHLHS- Forward handspring from the lunge and from a higher surface; FHHHS- Forward handspring from the hop and from a higher surface; FHPBS- Forward handspring from the push-off from the take-off board; FHPOM- Forward handspring from the push-off from the mat.

**Table 2:** Partameters for velocity

	**Forward handspring**		**QLKH**		**KHAWM**		**KHHWM**		**HH**		**HHLS**		**HHHS**	

	**X**	**Y**	**X**	**Y**	**X**	**Y**	**X**	**Y**	**X**	**Y**	**X**	**X**	**Y**	**X**

CG velocity after bounding step (cm/s)	289	−172	83	5	143	−37	110	−111	83	5	335	−154	365	−167
CG velocity during swing phase (m/s)	314	−44	167	−68	144	−64	199	−65	167	−68	342	−18	368	−79
CG velocity in first contact take-off leg (m/s)	314	−44	162	−65	144	−57	192	−65	162	−65	343	−53	368	−66
CG velocity in take-off phase (m/s)	300	55	184	52	147	63	187	54	184	52	317	80	341	48
CG velocity in first hand contact (m/s)	298	58	181	63	148	54	190	45	181	63	313	85	334	66
Cgvelocity in push-off phase (m/s)	278	66							122	123	245	168	293	138
CG velocity in max flight phase (m/s)	268	0									234	0	307	0
CG velocity at the moment of landing (m/s)	198	−97												

	**Forward handspring**		**BRIDGE**		**CUPB**		**FHLHS**		**FHHHS**		**FHPBS**		**FHPOM**	

	**X**	**Y**	**X**	**Y**	**X**	**Y**	**X**	**Y**	**X**	**Y**	**X**	**Y**	**X**	**Y**

CG velocity after bounding step (m/s)	289	−172					180	−58	134	−144	329	−155	321	−161
CG velocity during swing phase (m/s)	314	−44					183	−61	236	−33	330	−64	328	−51
CG velocity in first contact take-off leg (m/s)	314	−44					182	−59	208	−92	332	−52	328	−51
CG velocity in take-off phase (m/s)	300	55					193	62	234	58	310	92	310	80
CG velocity in first hand contact (m/s)	298	58	0	0			191	51	235	50	283	114	302	96
Cgvelocity in push-off phase (m/s)	278	66	22	0			184	28	226	57	228	191	220	89
CG velocity in max flight phase (m/s)	268	0	14	0			180	0	218	0	196	0	194	0
CG velocity at the moment of landing (m/s)	198	−97	68	25	144	−265	117	−182	171	−205	150	−200	137	−122

X - horizontal veloctiy; Y - vertical velocity; CG- center of gravity; QLKH- quick leg kick to handstand; KHAWM- Kicking to a handstand against a wall/upright mat; KHHWM- Kicking to a handstand from the hop against a wall/upright mat; HH- Handstand Hop; HHLS- Handstand Hop landing on the back on the lower surface; HHHS- Handstand Hop landing on the back on the higher surface; CUPB - clear underswing from parallel bars; BRIDGE- Drawing the co-gymnast over the back through the bridge; FHLHS-Forward handspring from the lunge and from a higher surface; FHHHS- Forward handspring from the hop and from a higher surface; FHPBS- Forward handspring from the push-off from the take-off board; FHPOM- Forward handspring from the push-off from the mat.
